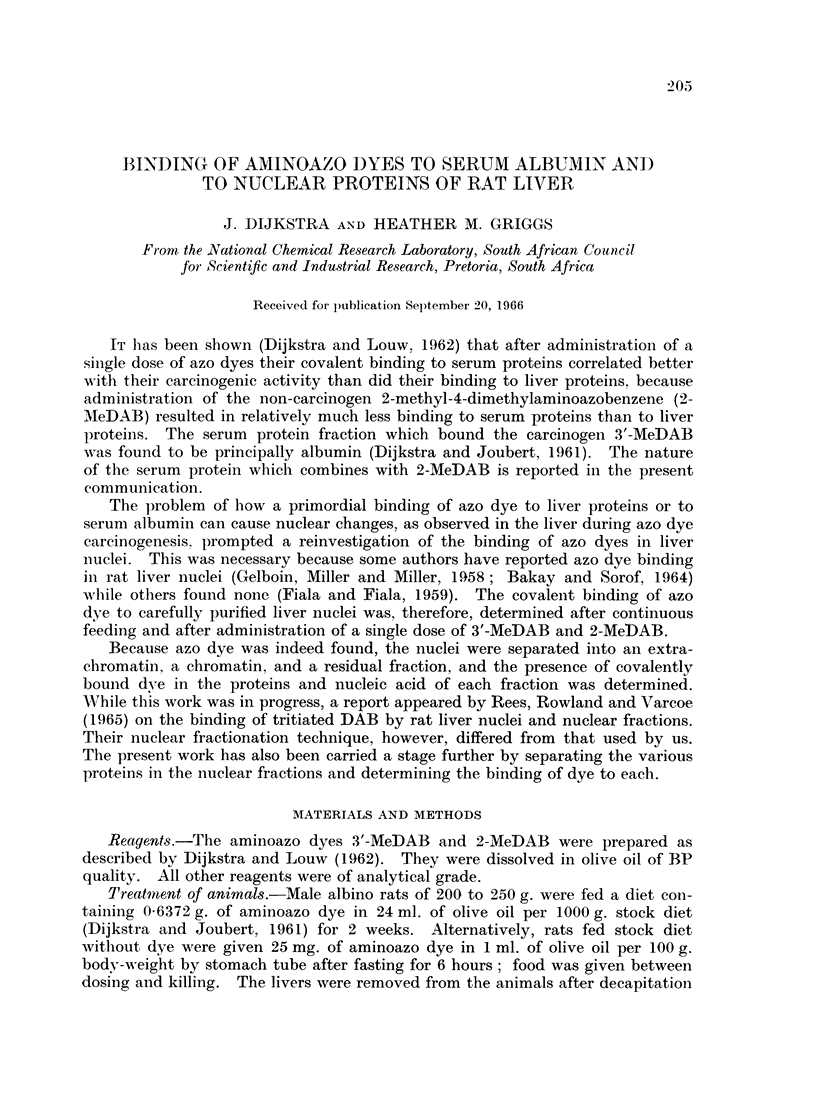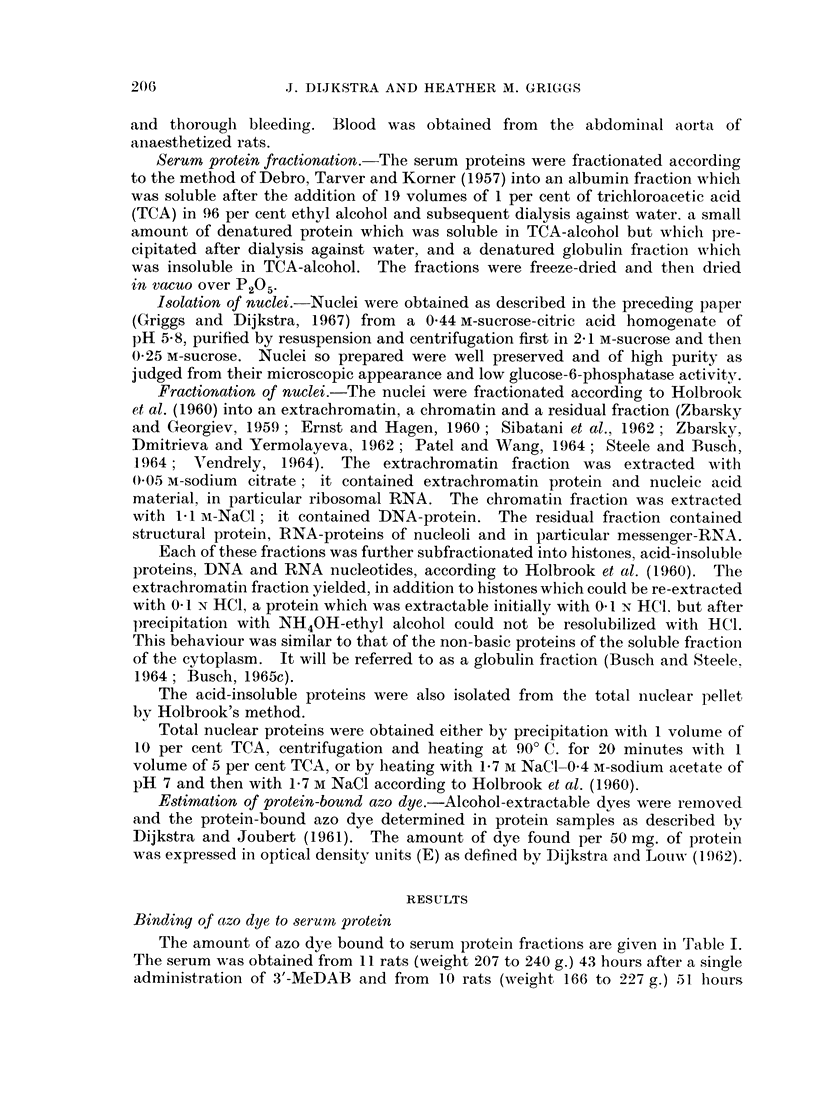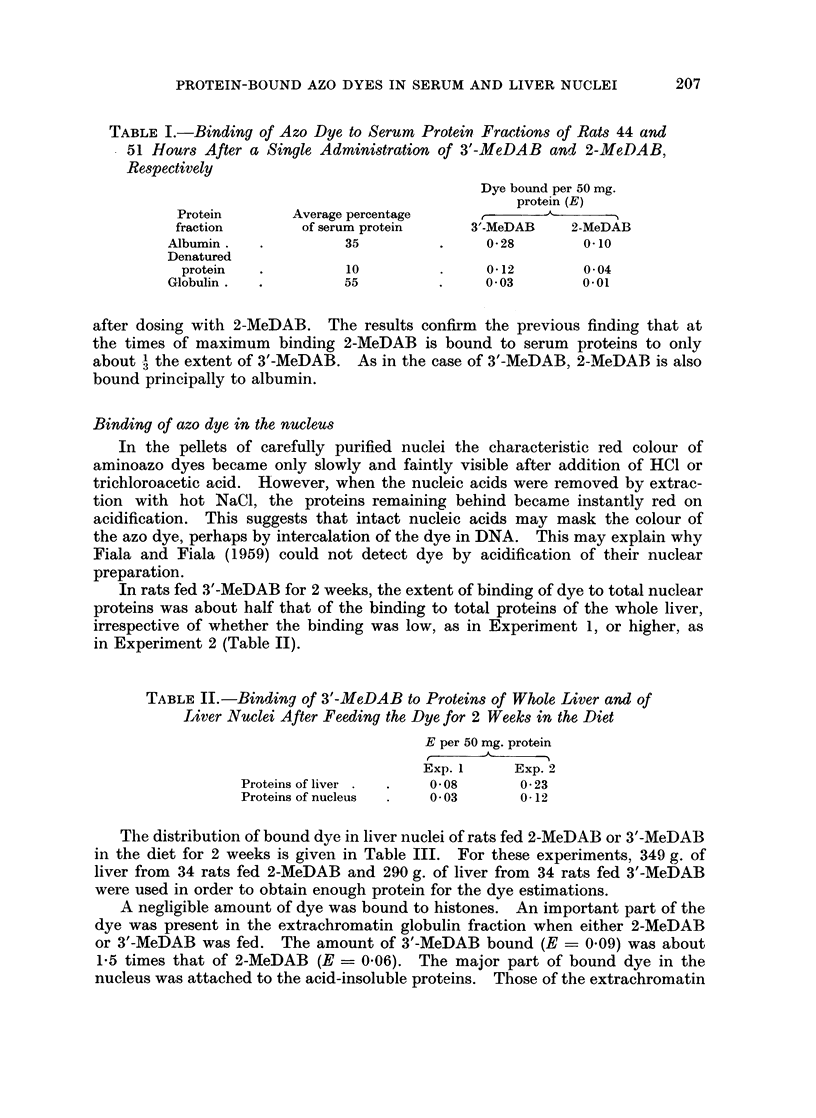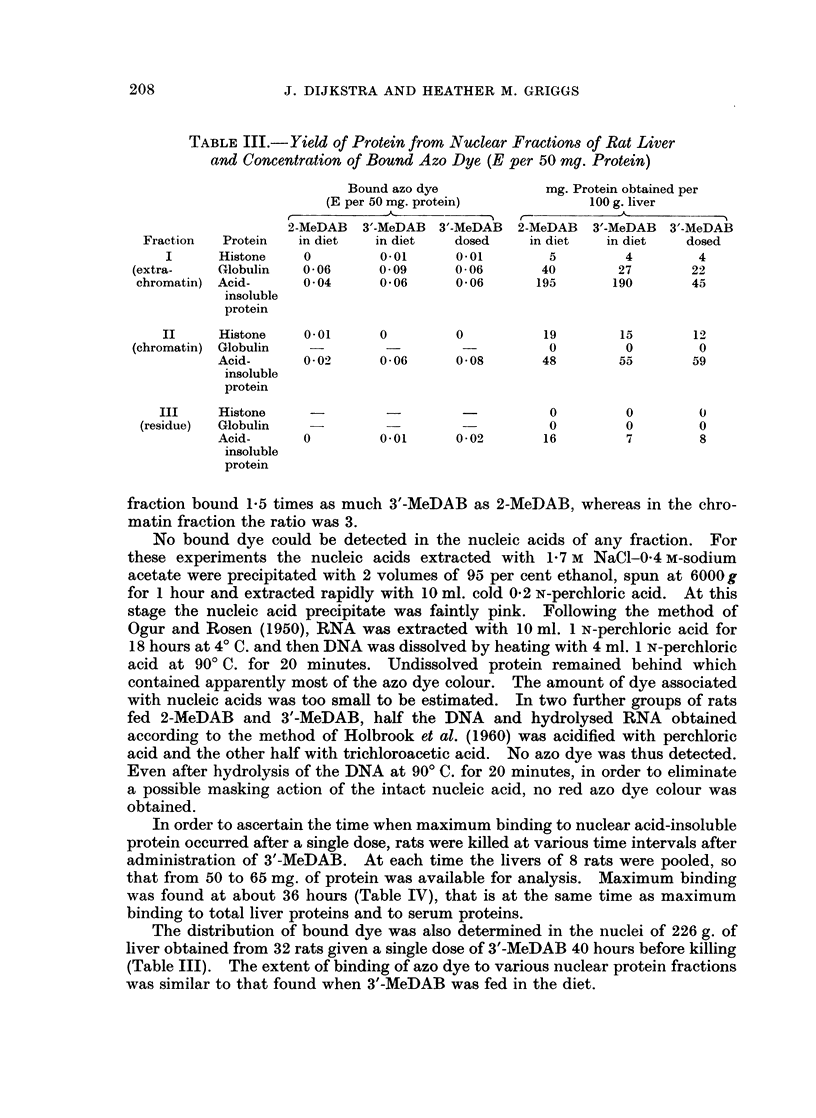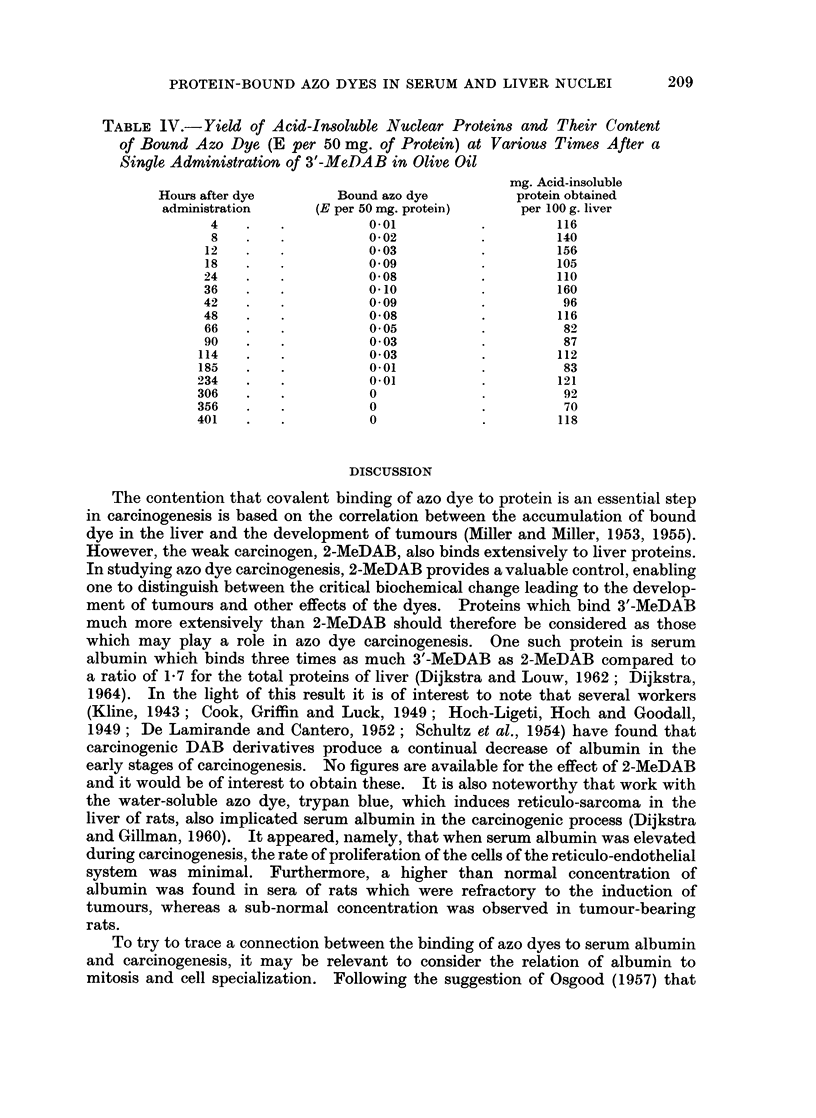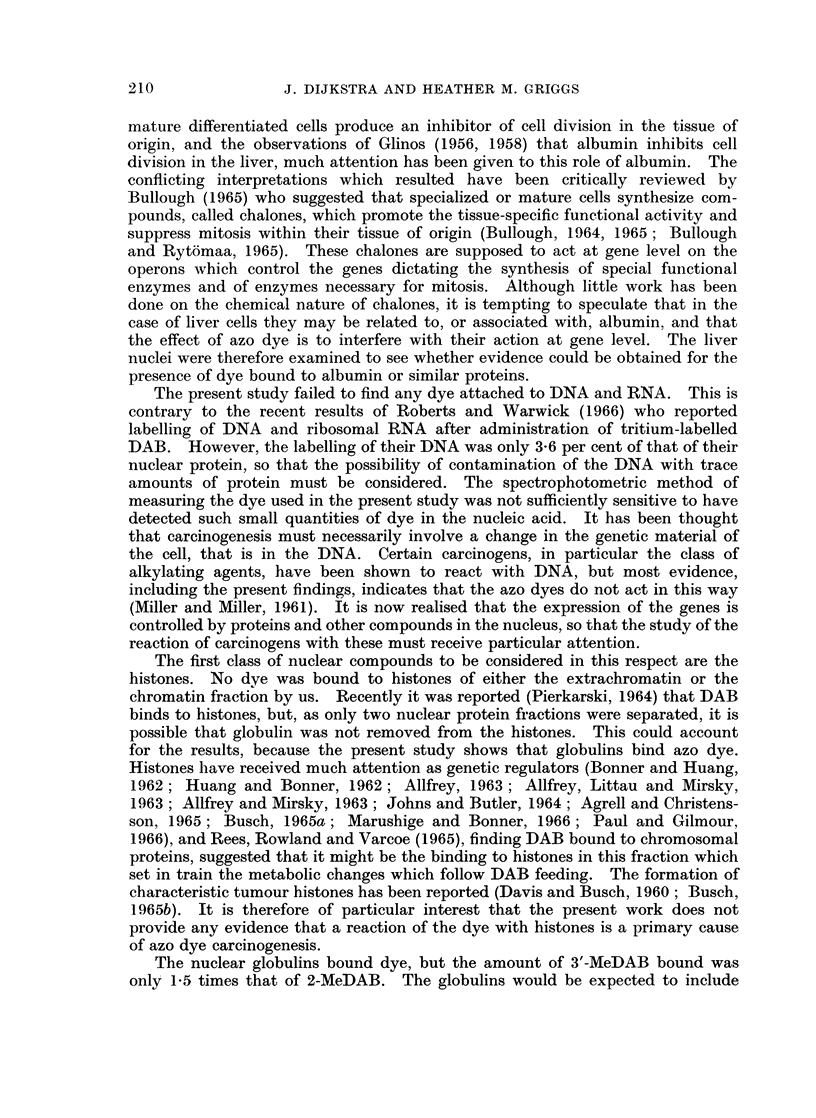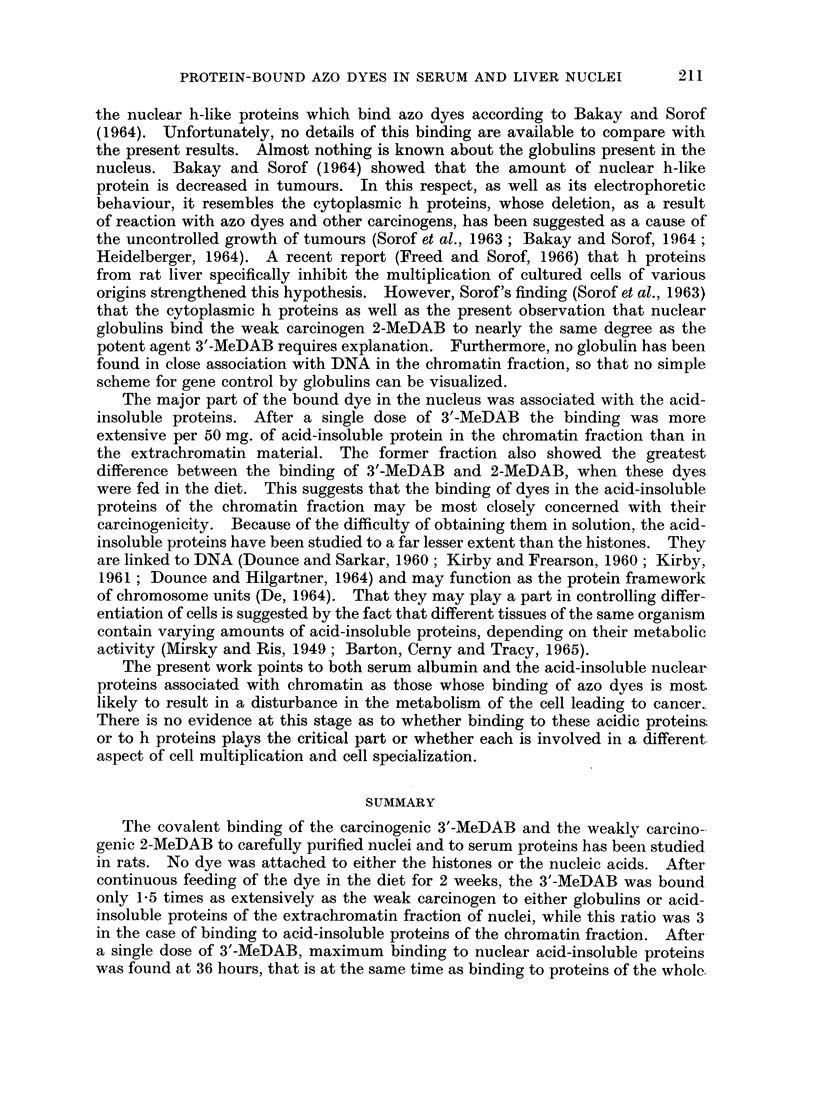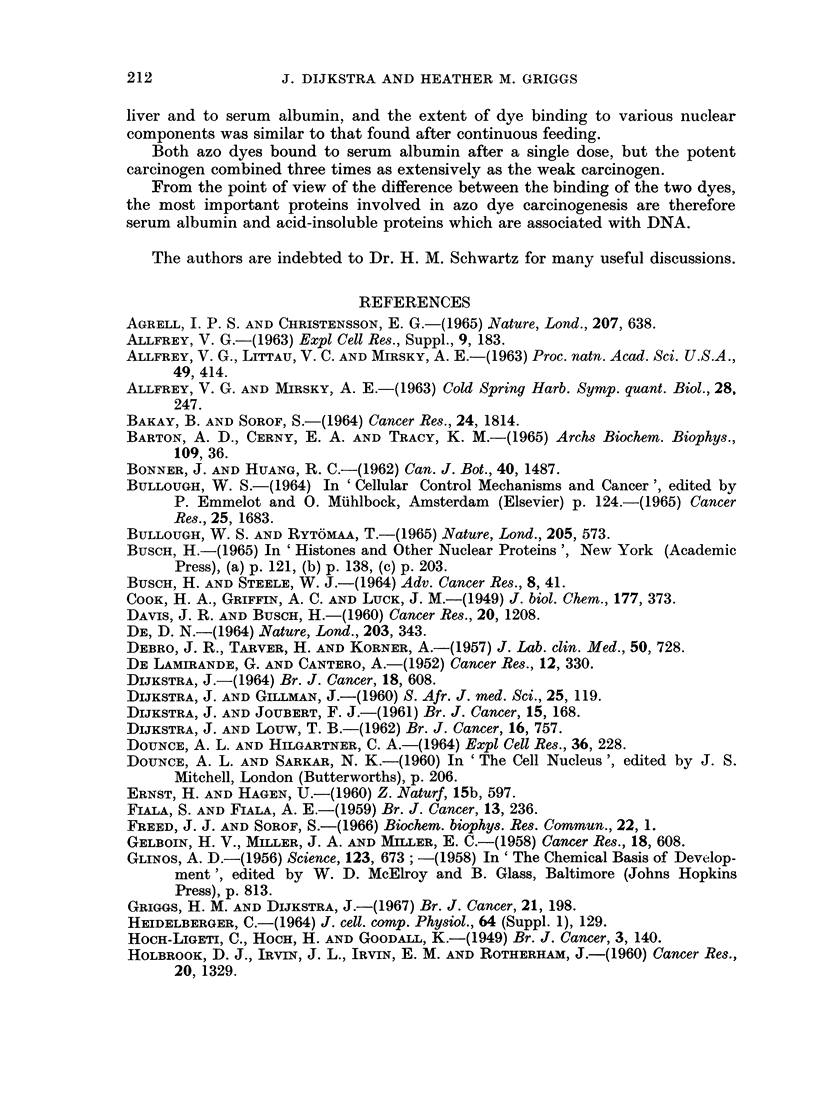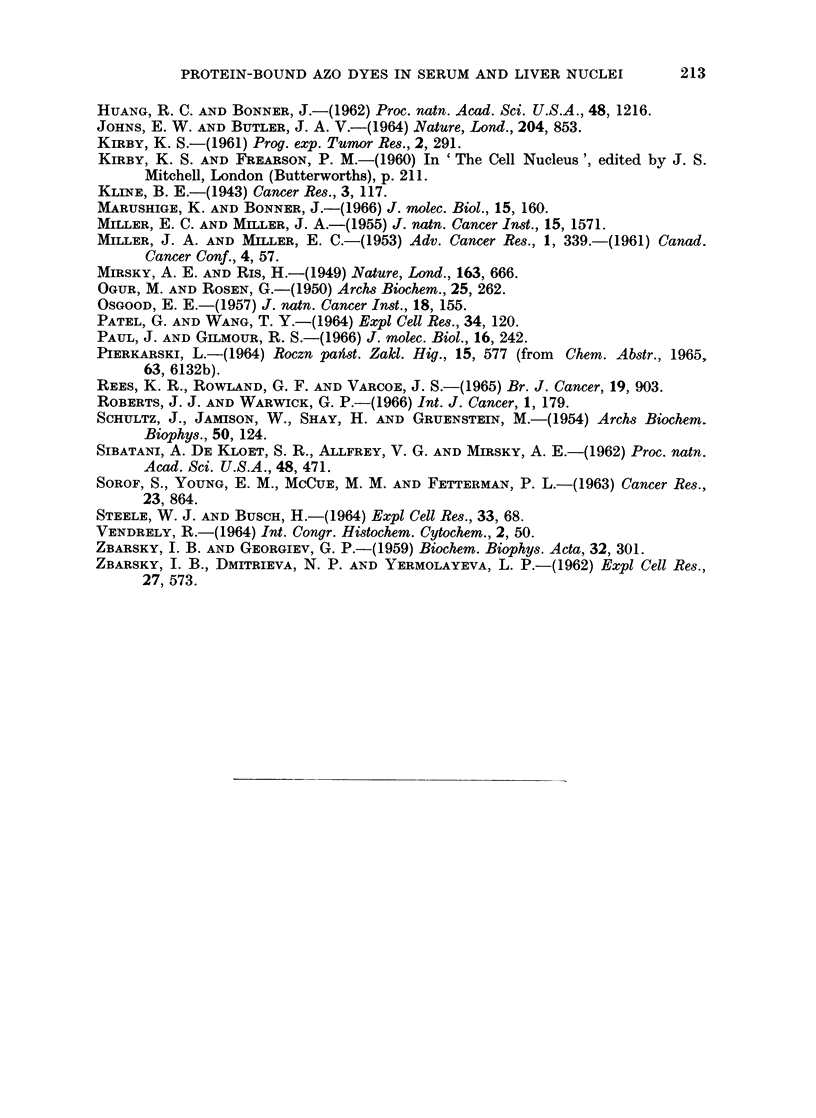# Binding of aminoazo dyes to serum albumin and to nuclear proteins of rat liver.

**DOI:** 10.1038/bjc.1967.22

**Published:** 1967-03

**Authors:** J. Dijkstra, H. M. Griggs


### BINDING OF AMINOAZO DYES TO SERUM ALBUMIN AND TO NUCLEAR PROTEINS OF RAT LIVER

J. DIJKSTRA AND HEATHER M. GRIGGS

*From the National Chemical Research Laboratory, South African Council for Scientific and Industrial Research, Pretoria, South Africa*

Received for publication September 20, 1966

It has been shown (Dijkstra and Louw, 1962) that after administration of a single dose of azo dyes their covalent binding to serum proteins correlated better with their carcinogenic activity than did their binding to liver proteins, because administration of the non-carcinogen 2-methyl-4-dimethylaminoazobenzene (2-MeDAB) resulted in relatively much less binding to serum proteins than to liver proteins. The serum protein fraction which bound the carcinogen 3′-MeDAB was found to be principally albumin (Dijkstra and Joubert, 1961). The nature of the serum protein which combines with 2-MeDAB is reported in the present communication.

The problem of how a primordial binding of azo dye to liver proteins or to serum albumin can cause nuclear changes, as observed in the liver during azo dye carcinogenesis, prompted a reinvestigation of the binding of azo dyes in liver nuclei. This was necessary because some authors have reported azo dye binding in rat liver nuclei (Gelboin, Miller and Miller, 1958 ; Bakay and Sorof, 1964) while others found none (Fiala and Fiala, 1959). The covalent binding of azo dye to carefully purified liver nuclei was, therefore, determined after continuous feeding and after administration of a single dose of 3′-MeDAB and 2-MeDAB.

Because azo dye was indeed found, the nuclei were separated into an extra-chromatin, a chromatin, and a residual fraction, and the presence of covalently bound dye in the proteins and nucleic acid of each fraction was determined. While this work was in progress, a report appeared by Rees, Rowland and Varcoe (1965) on the binding of tritiated DAB by rat liver nuclei and nuclear fractions. Their nuclear fractionation technique, however, differed from that used by us. The present work has also been carried a stage further by separating the various proteins in the nuclear fractions and determining the binding of dye to each.

### MATERIALS AND METHODS

*Reagents.*—The aminoazo dyes 3′-MeDAB and 2-MeDAB were prepared as described by Dijkstra and Louw (1962). They were dissolved in olive oil of BP quality. All other reagents were of analytical grade.

*Treatment of animals.*—Male albino rats of 200 to 250 g. were fed a diet containing 0·6372 g. of aminoazo dye in 24 ml. of olive oil per 1000 g. stock diet (Dijkstra and Joubert, 1961) for 2 weeks. Alternatively, rats fed stock diet without dye were given 25 mg. of aminoazo dye in 1 ml. of olive oil per 100 g. body-weight by stomach tube after fasting for 6 hours ; food was given between dosing and killing. The livers were removed from the animals after decapitation and thorough bleeding. Blood was obtained from the abdominal aorta of anaesthetized rats.

*Serum protein fractionation.*—The serum proteins were fractionated according to the method of Debro, Tarver and Korner (1957) into an albumin fraction which was soluble after the addition of 19 volumes of 1 per cent of trichloroacetic acid (TCA) in 96 per cent ethyl alcohol and subsequent dialysis against water, a small amount of denatured protein which was soluble in TCA-alcohol but which precipitated after dialysis against water, and a denatured globulin fraction which was insoluble in TCA-alcohol. The fractions were freeze-dried and then dried *in vacuo* over $P_2O_5$.

*Isolation of nuclei.*—Nuclei were obtained as described in the preceding paper (Griggs and Dijkstra, 1967) from a 0·44 M-sucrose-citric acid homogenate of pH 5·8, purified by resuspension and centrifugation first in 2·1 M-sucrose and then 0·25 M-sucrose. Nuclei so prepared were well preserved and of high purity as judged from their microscopic appearance and low glucose-6-phosphatase activity.

*Fractionation of nuclei.*—The nuclei were fractionated according to Holbrook *et al.* (1960) into an extrachromatin, a chromatin and a residual fraction (Zbarsky and Georgiev, 1959; Ernst and Hagen, 1960; Sibatani *et al.*, 1962; Zbarsky, Dmitrieva and Yermolayeva, 1962; Patel and Wang, 1964; Steele and Busch, 1964; Vendrely, 1964). The extrachromatin fraction was extracted with 0·05 M-sodium citrate; it contained extrachromatin protein and nucleic acid material, in particular ribosomal RNA. The chromatin fraction was extracted with 1·1 M-NaCl; it contained DNA-protein. The residual fraction contained structural protein, RNA-proteins of nucleoli and in particular messenger-RNA.

Each of these fractions was further subfractionated into histones, acid-insoluble proteins, DNA and RNA nucleotides, according to Holbrook *et al.* (1960). The extrachromatin fraction yielded, in addition to histones which could be re-extracted with 0·1 N HCl, a protein which was extractable initially with 0·1 N HCl, but after precipitation with $NH_4OH$-ethyl alcohol could not be resolubilized with HCl. This behaviour was similar to that of the non-basic proteins of the soluble fraction of the cytoplasm. It will be referred to as a globulin fraction (Busch and Steele, 1964; Busch, 1965*c*).

The acid-insoluble proteins were also isolated from the total nuclear pellet by Holbrook's method.

Total nuclear proteins were obtained either by precipitation with 1 volume of 10 per cent TCA, centrifugation and heating at 90° C. for 20 minutes with 1 volume of 5 per cent TCA, or by heating with 1·7 M NaCl–0·4 M-sodium acetate of pH 7 and then with 1·7 M NaCl according to Holbrook *et al.* (1960).

*Estimation of protein-bound azo dye.*—Alcohol-extractable dyes were removed and the protein-bound azo dye determined in protein samples as described by Dijkstra and Joubert (1961). The amount of dye found per 50 mg. of protein was expressed in optical density units (E) as defined by Dijkstra and Louw (1962).

### RESULTS

### *Binding of azo dye to serum protein*

The amount of azo dye bound to serum protein fractions are given in Table I. The serum was obtained from 11 rats (weight 207 to 240 g.) 43 hours after a single administration of 3′-MeDAB and from 10 rats (weight 166 to 227 g.) 51 hours

TABLE I.—*Binding of Azo Dye to Serum Protein Fractions of Rats 44 and 51 Hours After a Single Administration of 3′-MeDAB and 2-MeDAB, Respectively*

| Protein fraction | Average percentage of serum protein | Dye bound per 50 mg. protein (*E*) | |
|---|---|---|---|
| | | 3′-MeDAB | 2-MeDAB |
| Albumin | 35 | 0·28 | 0·10 |
| Denatured protein | 10 | 0·12 | 0·04 |
| Globulin | 55 | 0·03 | 0·01 |

after dosing with 2-MeDAB. The results confirm the previous finding that at the times of maximum binding 2-MeDAB is bound to serum proteins to only about $\frac{1}{3}$ the extent of 3′-MeDAB. As in the case of 3′-MeDAB, 2-MeDAB is also bound principally to albumin.

*Binding of azo dye in the nucleus*

In the pellets of carefully purified nuclei the characteristic red colour of aminoazo dyes became only slowly and faintly visible after addition of HCl or trichloroacetic acid. However, when the nucleic acids were removed by extraction with hot NaCl, the proteins remaining behind became instantly red on acidification. This suggests that intact nucleic acids may mask the colour of the azo dye, perhaps by intercalation of the dye in DNA. This may explain why Fiala and Fiala (1959) could not detect dye by acidification of their nuclear preparation.

In rats fed 3′-MeDAB for 2 weeks, the extent of binding of dye to total nuclear proteins was about half that of the binding to total proteins of the whole liver, irrespective of whether the binding was low, as in Experiment 1, or higher, as in Experiment 2 (Table II).

TABLE II.—*Binding of 3′-MeDAB to Proteins of Whole Liver and of Liver Nuclei After Feeding the Dye for 2 Weeks in the Diet*

| | *E* per 50 mg. protein | |
|---|---|---|
| | Exp. 1 | Exp. 2 |
| Proteins of liver | 0·08 | 0·23 |
| Proteins of nucleus | 0·03 | 0·12 |

The distribution of bound dye in liver nuclei of rats fed 2-MeDAB or 3′-MeDAB in the diet for 2 weeks is given in Table III. For these experiments, 349 g. of liver from 34 rats fed 2-MeDAB and 290 g. of liver from 34 rats fed 3′-MeDAB were used in order to obtain enough protein for the dye estimations.

A negligible amount of dye was bound to histones. An important part of the dye was present in the extrachromatin globulin fraction when either 2-MeDAB or 3′-MeDAB was fed. The amount of 3′-MeDAB bound ($E = 0{\cdot}09$) was about 1·5 times that of 2-MeDAB ($E = 0{\cdot}06$). The major part of bound dye in the nucleus was attached to the acid-insoluble proteins. Those of the extrachromatin

TABLE III.—*Yield of Protein from Nuclear Fractions of Rat Liver and Concentration of Bound Azo Dye (E per 50 mg. Protein)*

| Fraction | Protein | Bound azo dye (E per 50 mg. protein) 2-MeDAB in diet | 3′-MeDAB in diet | 3′-MeDAB dosed | mg. Protein obtained per 100 g. liver 2-MeDAB in diet | 3′-MeDAB in diet | 3′-MeDAB dosed |
|---|---|---|---|---|---|---|---|
| I | Histone | 0 | 0·01 | 0·01 | 5 | 4 | 4 |
| (extra- | Globulin | 0·06 | 0·09 | 0·06 | 40 | 27 | 22 |
| chromatin) | Acid-insoluble protein | 0·04 | 0·06 | 0·06 | 195 | 190 | 45 |
| II | Histone | 0·01 | 0 | 0 | 19 | 15 | 12 |
| (chromatin) | Globulin | — | — | — | 0 | 0 | 0 |
| | Acid-insoluble protein | 0·02 | 0·06 | 0·08 | 48 | 55 | 59 |
| III | Histone | — | — | — | 0 | 0 | 0 |
| (residue) | Globulin | — | — | — | 0 | 0 | 0 |
| | Acid-insoluble protein | 0 | 0·01 | 0·02 | 16 | 7 | 8 |

fraction bound 1·5 times as much 3′-MeDAB as 2-MeDAB, whereas in the chromatin fraction the ratio was 3.

No bound dye could be detected in the nucleic acids of any fraction. For these experiments the nucleic acids extracted with 1·7 M NaCl–0·4 M-sodium acetate were precipitated with 2 volumes of 95 per cent ethanol, spun at 6000 *g* for 1 hour and extracted rapidly with 10 ml. cold 0·2 N-perchloric acid. At this stage the nucleic acid precipitate was faintly pink. Following the method of Ogur and Rosen (1950), RNA was extracted with 10 ml. 1 N-perchloric acid for 18 hours at 4° C. and then DNA was dissolved by heating with 4 ml. 1 N-perchloric acid at 90° C. for 20 minutes. Undissolved protein remained behind which contained apparently most of the azo dye colour. The amount of dye associated with nucleic acids was too small to be estimated. In two further groups of rats fed 2-MeDAB and 3′-MeDAB, half the DNA and hydrolysed RNA obtained according to the method of Holbrook *et al.* (1960) was acidified with perchloric acid and the other half with trichloroacetic acid. No azo dye was thus detected. Even after hydrolysis of the DNA at 90° C. for 20 minutes, in order to eliminate a possible masking action of the intact nucleic acid, no red azo dye colour was obtained.

In order to ascertain the time when maximum binding to nuclear acid-insoluble protein occurred after a single dose, rats were killed at various time intervals after administration of 3′-MeDAB. At each time the livers of 8 rats were pooled, so that from 50 to 65 mg. of protein was available for analysis. Maximum binding was found at about 36 hours (Table IV), that is at the same time as maximum binding to total liver proteins and to serum proteins.

The distribution of bound dye was also determined in the nuclei of 226 g. of liver obtained from 32 rats given a single dose of 3′-MeDAB 40 hours before killing (Table III). The extent of binding of azo dye to various nuclear protein fractions was similar to that found when 3′-MeDAB was fed in the diet.

TABLE IV.—*Yield of Acid-Insoluble Nuclear Proteins and Their Content of Bound Azo Dye* (E *per* 50 mg. *of Protein*) *at Various Times After a Single Administration of* 3′-*MeDAB in Olive Oil*

| Hours after dye administration | Bound azo dye (*E* per 50 mg. protein) | mg. Acid-insoluble protein obtained per 100 g. liver |
|---|---|---|
| 4 | 0·01 | 116 |
| 8 | 0·02 | 140 |
| 12 | 0·03 | 156 |
| 18 | 0·09 | 105 |
| 24 | 0·08 | 110 |
| 36 | 0·10 | 160 |
| 42 | 0·09 | 96 |
| 48 | 0·08 | 116 |
| 66 | 0·05 | 82 |
| 90 | 0·03 | 87 |
| 114 | 0·03 | 112 |
| 185 | 0·01 | 83 |
| 234 | 0·01 | 121 |
| 306 | 0 | 92 |
| 356 | 0 | 70 |
| 401 | 0 | 118 |

### DISCUSSION

The contention that covalent binding of azo dye to protein is an essential step in carcinogenesis is based on the correlation between the accumulation of bound dye in the liver and the development of tumours (Miller and Miller, 1953, 1955). However, the weak carcinogen, 2-MeDAB, also binds extensively to liver proteins. In studying azo dye carcinogenesis, 2-MeDAB provides a valuable control, enabling one to distinguish between the critical biochemical change leading to the development of tumours and other effects of the dyes. Proteins which bind 3′-MeDAB much more extensively than 2-MeDAB should therefore be considered as those which may play a role in azo dye carcinogenesis. One such protein is serum albumin which binds three times as much 3′-MeDAB as 2-MeDAB compared to a ratio of 1·7 for the total proteins of liver (Dijkstra and Louw, 1962 ; Dijkstra, 1964). In the light of this result it is of interest to note that several workers (Kline, 1943 ; Cook, Griffin and Luck, 1949 ; Hoch-Ligeti, Hoch and Goodall, 1949 ; De Lamirande and Cantero, 1952 ; Schultz *et al.*, 1954) have found that carcinogenic DAB derivatives produce a continual decrease of albumin in the early stages of carcinogenesis. No figures are available for the effect of 2-MeDAB and it would be of interest to obtain these. It is also noteworthy that work with the water-soluble azo dye, trypan blue, which induces reticulo-sarcoma in the liver of rats, also implicated serum albumin in the carcinogenic process (Dijkstra and Gillman, 1960). It appeared, namely, that when serum albumin was elevated during carcinogenesis, the rate of proliferation of the cells of the reticulo-endothelial system was minimal. Furthermore, a higher than normal concentration of albumin was found in sera of rats which were refractory to the induction of tumours, whereas a sub-normal concentration was observed in tumour-bearing rats.

To try to trace a connection between the binding of azo dyes to serum albumin and carcinogenesis, it may be relevant to consider the relation of albumin to mitosis and cell specialization. Following the suggestion of Osgood (1957) that mature differentiated cells produce an inhibitor of cell division in the tissue of origin, and the observations of Glinos (1956, 1958) that albumin inhibits cell division in the liver, much attention has been given to this role of albumin. The conflicting interpretations which resulted have been critically reviewed by Bullough (1965) who suggested that specialized or mature cells synthesize compounds, called chalones, which promote the tissue-specific functional activity and suppress mitosis within their tissue of origin (Bullough, 1964, 1965; Bullough and Rytömaa, 1965). These chalones are supposed to act at gene level on the operons which control the genes dictating the synthesis of special functional enzymes and of enzymes necessary for mitosis. Although little work has been done on the chemical nature of chalones, it is tempting to speculate that in the case of liver cells they may be related to, or associated with, albumin, and that the effect of azo dye is to interfere with their action at gene level. The liver nuclei were therefore examined to see whether evidence could be obtained for the presence of dye bound to albumin or similar proteins.

The present study failed to find any dye attached to DNA and RNA. This is contrary to the recent results of Roberts and Warwick (1966) who reported labelling of DNA and ribosomal RNA after administration of tritium-labelled DAB. However, the labelling of their DNA was only 3·6 per cent of that of their nuclear protein, so that the possibility of contamination of the DNA with trace amounts of protein must be considered. The spectrophotometric method of measuring the dye used in the present study was not sufficiently sensitive to have detected such small quantities of dye in the nucleic acid. It has been thought that carcinogenesis must necessarily involve a change in the genetic material of the cell, that is in the DNA. Certain carcinogens, in particular the class of alkylating agents, have been shown to react with DNA, but most evidence, including the present findings, indicates that the azo dyes do not act in this way (Miller and Miller, 1961). It is now realised that the expression of the genes is controlled by proteins and other compounds in the nucleus, so that the study of the reaction of carcinogens with these must receive particular attention.

The first class of nuclear compounds to be considered in this respect are the histones. No dye was bound to histones of either the extrachromatin or the chromatin fraction by us. Recently it was reported (Pierkarski, 1964) that DAB binds to histones, but, as only two nuclear protein fractions were separated, it is possible that globulin was not removed from the histones. This could account for the results, because the present study shows that globulins bind azo dye. Histones have received much attention as genetic regulators (Bonner and Huang, 1962; Huang and Bonner, 1962; Allfrey, 1963; Allfrey, Littau and Mirsky, 1963; Allfrey and Mirsky, 1963; Johns and Butler, 1964; Agrell and Christensson, 1965; Busch, 1965*a*; Marushige and Bonner, 1966; Paul and Gilmour, 1966), and Rees, Rowland and Varcoe (1965), finding DAB bound to chromosomal proteins, suggested that it might be the binding to histones in this fraction which set in train the metabolic changes which follow DAB feeding. The formation of characteristic tumour histones has been reported (Davis and Busch, 1960; Busch, 1965*b*). It is therefore of particular interest that the present work does not provide any evidence that a reaction of the dye with histones is a primary cause of azo dye carcinogenesis.

The nuclear globulins bound dye, but the amount of 3′-MeDAB bound was only 1·5 times that of 2-MeDAB. The globulins would be expected to include the nuclear h-like proteins which bind azo dyes according to Bakay and Sorof (1964). Unfortunately, no details of this binding are available to compare with the present results. Almost nothing is known about the globulins present in the nucleus. Bakay and Sorof (1964) showed that the amount of nuclear h-like protein is decreased in tumours. In this respect, as well as its electrophoretic behaviour, it resembles the cytoplasmic h proteins, whose deletion, as a result of reaction with azo dyes and other carcinogens, has been suggested as a cause of the uncontrolled growth of tumours (Sorof *et al.*, 1963 ; Bakay and Sorof, 1964 ; Heidelberger, 1964). A recent report (Freed and Sorof, 1966) that h proteins from rat liver specifically inhibit the multiplication of cultured cells of various origins strengthened this hypothesis. However, Sorof's finding (Sorof *et al.*, 1963) that the cytoplasmic h proteins as well as the present observation that nuclear globulins bind the weak carcinogen 2-MeDAB to nearly the same degree as the potent agent 3′-MeDAB requires explanation. Furthermore, no globulin has been found in close association with DNA in the chromatin fraction, so that no simple scheme for gene control by globulins can be visualized.

The major part of the bound dye in the nucleus was associated with the acid-insoluble proteins. After a single dose of 3′-MeDAB the binding was more extensive per 50 mg. of acid-insoluble protein in the chromatin fraction than in the extrachromatin material. The former fraction also showed the greatest difference between the binding of 3′-MeDAB and 2-MeDAB, when these dyes were fed in the diet. This suggests that the binding of dyes in the acid-insoluble proteins of the chromatin fraction may be most closely concerned with their carcinogenicity. Because of the difficulty of obtaining them in solution, the acid-insoluble proteins have been studied to a far lesser extent than the histones. They are linked to DNA (Dounce and Sarkar, 1960 ; Kirby and Frearson, 1960 ; Kirby, 1961 ; Dounce and Hilgartner, 1964) and may function as the protein framework of chromosome units (De, 1964). That they may play a part in controlling differentiation of cells is suggested by the fact that different tissues of the same organism contain varying amounts of acid-insoluble proteins, depending on their metabolic activity (Mirsky and Ris, 1949 ; Barton, Cerny and Tracy, 1965).

The present work points to both serum albumin and the acid-insoluble nuclear proteins associated with chromatin as those whose binding of azo dyes is most likely to result in a disturbance in the metabolism of the cell leading to cancer. There is no evidence at this stage as to whether binding to these acidic proteins or to h proteins plays the critical part or whether each is involved in a different aspect of cell multiplication and cell specialization.

### SUMMARY

The covalent binding of the carcinogenic 3′-MeDAB and the weakly carcinogenic 2-MeDAB to carefully purified nuclei and to serum proteins has been studied in rats. No dye was attached to either the histones or the nucleic acids. After continuous feeding of the dye in the diet for 2 weeks, the 3′-MeDAB was bound only 1·5 times as extensively as the weak carcinogen to either globulins or acid-insoluble proteins of the extrachromatin fraction of nuclei, while this ratio was 3 in the case of binding to acid-insoluble proteins of the chromatin fraction. After a single dose of 3′-MeDAB, maximum binding to nuclear acid-insoluble proteins was found at 36 hours, that is at the same time as binding to proteins of the whole liver and to serum albumin, and the extent of dye binding to various nuclear components was similar to that found after continuous feeding.

Both azo dyes bound to serum albumin after a single dose, but the potent carcinogen combined three times as extensively as the weak carcinogen.

From the point of view of the difference between the binding of the two dyes, the most important proteins involved in azo dye carcinogenesis are therefore serum albumin and acid-insoluble proteins which are associated with DNA.

The authors are indebted to Dr. H. M. Schwartz for many useful discussions.

### REFERENCES

AGRELL, I. P. S. AND CHRISTENSSON, E. G.—(1965) *Nature, Lond.*, **207**, 638.

ALLFREY, V. G.—(1963) *Expl Cell Res.*, Suppl., **9**, 183.

ALLFREY, V. G., LITTAU, V. C. AND MIRSKY, A. E.—(1963) *Proc. natn. Acad. Sci. U.S.A.*, **49**, 414.

ALLFREY, V. G. AND MIRSKY, A. E.—(1963) *Cold Spring Harb. Symp. quant. Biol.*, **28**, 247.

BAKAY, B. AND SOROF, S.—(1964) *Cancer Res.*, **24**, 1814.

BARTON, A. D., CERNY, E. A. AND TRACY, K. M.—(1965) *Archs Biochem. Biophys.*, **109**, 36.

BONNER, J. AND HUANG, R. C.—(1962) *Can. J. Bot.*, **40**, 1487.

BULLOUGH, W. S.—(1964) In ' Cellular Control Mechanisms and Cancer ', edited by P. Emmelot and O. Mühlbock, Amsterdam (Elsevier) p. 124.—(1965) *Cancer Res.*, **25**, 1683.

BULLOUGH, W. S. AND RYTÖMAA, T.—(1965) *Nature, Lond.*, **205**, 573.

BUSCH, H.—(1965) In ' Histones and Other Nuclear Proteins ', New York (Academic Press), (a) p. 121, (b) p. 138, (c) p. 203.

BUSCH, H. AND STEELE, W. J.—(1964) *Adv. Cancer Res.*, **8**, 41.

COOK, H. A., GRIFFIN, A. C. AND LUCK, J. M.—(1949) *J. biol. Chem.*, **177**, 373.

DAVIS, J. R. AND BUSCH, H.—(1960) *Cancer Res.*, **20**, 1208.

DE, D. N.—(1964) *Nature, Lond.*, **203**, 343.

DEBRO, J. R., TARVER, H. AND KORNER, A.—(1957) *J. Lab. clin. Med.*, **50**, 728.

DE LAMIRANDE, G. AND CANTERO, A.—(1952) *Cancer Res.*, **12**, 330.

DIJKSTRA, J.—(1964) *Br. J. Cancer*, **18**, 608.

DIJKSTRA, J. AND GILLMAN, J.—(1960) *S. Afr. J. med. Sci.*, **25**, 119.

DIJKSTRA, J. AND JOUBERT, F. J.—(1961) *Br. J. Cancer*, **15**, 168.

DIJKSTRA, J. AND LOUW, T. B.—(1962) *Br. J. Cancer*, **16**, 757.

DOUNCE, A. L. AND HILGARTNER, C. A.—(1964) *Expl Cell Res.*, **36**, 228.

DOUNCE, A. L. AND SARKAR, N. K.—(1960) In ' The Cell Nucleus ', edited by J. S. Mitchell, London (Butterworths), p. 206.

ERNST, H. AND HAGEN, U.—(1960) *Z. Naturf*, **15b**, 597.

FIALA, S. AND FIALA, A. E.—(1959) *Br. J. Cancer*, **13**, 236.

FREED, J. J. AND SOROF, S.—(1966) *Biochem. biophys. Res. Commun.*, **22**, 1.

GELBOIN, H. V., MILLER, J. A. AND MILLER, E. C.—(1958) *Cancer Res.*, **18**, 608.

GLINOS, A. D.—(1956) *Science*, **123**, 673 ; —(1958) In ' The Chemical Basis of Development ', edited by W. D. McElroy and B. Glass, Baltimore (Johns Hopkins Press), p. 813.

GRIGGS, H. M. AND DIJKSTRA, J.—(1967) *Br. J. Cancer*, **21**, 198.

HEIDELBERGER, C.—(1964) *J. cell. comp. Physiol.*, **64** (Suppl. 1), 129.

HOCH-LIGETI, C., HOCH, H. AND GOODALL, K.—(1949) *Br. J. Cancer*, **3**, 140.

HOLBROOK, D. J., IRVIN, J. L., IRVIN, E. M. AND ROTHERHAM, J.—(1960) *Cancer Res.*, **20**, 1329.

HUANG, R. C. AND BONNER, J.—(1962) *Proc. natn. Acad. Sci. U.S.A.*, **48**, 1216.
JOHNS, E. W. AND BUTLER, J. A. V.—(1964) *Nature, Lond.*, **204**, 853.
KIRBY, K. S.—(1961) *Prog. exp. Tumor Res.*, **2**, 291.
KIRBY, K. S. AND FREARSON, P. M.—(1960) In ' The Cell Nucleus ', edited by J. S. Mitchell, London (Butterworths), p. 211.
KLINE, B. E.—(1943) *Cancer Res.*, **3**, 117.
MARUSHIGE, K. AND BONNER, J.—(1966) *J. molec. Biol.*, **15**, 160.
MILLER, E. C. AND MILLER, J. A.—(1955) *J. natn. Cancer Inst.*, **15**, 1571.
MILLER, J. A. AND MILLER, E. C.—(1953) *Adv. Cancer Res.*, **1**, 339.—(1961) *Canad. Cancer Conf.*, **4**, 57.
MIRSKY, A. E. AND RIS, H.—(1949) *Nature, Lond.*, **163**, 666.
OGUR, M. AND ROSEN, G.—(1950) *Archs Biochem.*, **25**, 262.
OSGOOD, E. E.—(1957) *J. natn. Cancer Inst.*, **18**, 155.
PATEL, G. AND WANG, T. Y.—(1964) *Expl Cell Res.*, **34**, 120.
PAUL, J. AND GILMOUR, R. S.—(1966) *J. molec. Biol.*, **16**, 242.
PIERKARSKI, L.—(1964) *Roczn państ. Zakl. Hig.*, **15**, 577 (from *Chem. Abstr.*, 1965, **63**, 6132b).
REES, K. R., ROWLAND, G. F. AND VARCOE, J. S.—(1965) *Br. J. Cancer*, **19**, 903.
ROBERTS, J. J. AND WARWICK, G. P.—(1966) *Int. J. Cancer*, **1**, 179.
SCHULTZ, J., JAMISON, W., SHAY, H. AND GRUENSTEIN, M.—(1954) *Archs Biochem. Biophys.*, **50**, 124.
SIBATANI, A. DE KLOET, S. R., ALLFREY, V. G. AND MIRSKY, A. E.—(1962) *Proc. natn. Acad. Sci. U.S.A.*, **48**, 471.
SOROF, S., YOUNG, E. M., MCCUE, M. M. AND FETTERMAN, P. L.—(1963) *Cancer Res.*, **23**, 864.
STEELE, W. J. AND BUSCH, H.—(1964) *Expl Cell Res.*, **33**, 68.
VENDRELY, R.—(1964) *Int. Congr. Histochem. Cytochem.*, **2**, 50.
ZBARSKY, I. B. AND GEORGIEV, G. P.—(1959) *Biochem. Biophys. Acta*, **32**, 301.
ZBARSKY, I. B., DMITRIEVA, N. P. AND YERMOLAYEVA, L. P.—(1962) *Expl Cell Res.*, **27**, 573.